# Impact of scheduled cesarean sections on non-working days on maternal and neonatal outcomes: a retrospective cohort study based on propensity score matching

**DOI:** 10.3389/fmed.2026.1839582

**Published:** 2026-07-14

**Authors:** Ting Fang Tan, Qing Fang Wei, Jian Chun Huang, Kai Sun Zhao

**Affiliations:** Department of Obstetrics, The Third Affiliated Hospital of Guangxi Medical University, The Second Nanning People's Hospital, Nanning, Guangxi, China

**Keywords:** scheduled cesarean sections, non-working days, propensity score matching, maternal and neonatal outcomes, retrospective cohort study

## Abstract

**Objective:**

This study aimed to investigate the impact of performing scheduled cesarean sections for term singleton pregnancies on working versus non-working days on maternal and neonatal outcomes.

**Methods:**

This retrospective study included women who underwent scheduled cesarean sections for term singleton pregnancies at our hospital between January and December 2024. Participants were divided into working-day and non-working-day groups based on the date of surgery. Propensity score matching (PSM) (1:1 nearest neighbor matching, caliper width 0.2 times the propensity score standard deviation) balanced confounding factors, including maternal age, gestational age, parity, prior cesarean deliveries, conception method, hypertensive disorders, gestational diabetes, and pre-pregnancy body mass index (BMI). After matching, we compared postoperative fever, neonatal transfer, surgery-related outcomes, and umbilical cord blood gas parameters between the groups. Multivariable linear regression analysis was additionally performed to adjust for residual confounding. Staffing configurations for obstetrics, anesthesia, neonatology, and nursing during working and non-working days are detailed in the Methods section.

**Results:**

Among the 300 women enrolled, 100 remained in each group after matching (all standardized mean differences <0.1). Matched analysis revealed no significant between-group differences in postoperative fever, neonatal transfer, surgical blood loss, postoperative hospital stay, antibiotic duration, or umbilical cord pH and lactate levels (*p* > 0.05). Multivariable regression analysis showed that surgery on non-working day**s** was independently associated with a shorter operative duration (*β* = −3.82, 95% CI: −7.22 to −0.42, *p* = 0.029), without significant differences in other outcomes.

**Conclusion:**

In this single-center setting with standardized staffing, no statistically significant differences in short-term adverse maternal or neonatal outcomes were detected between scheduled cesarean sections performed on working and non-working days in this study population and based on the available sample size.

## Introduction

Cesarean section is a critical delivery mode for managing high-risk pregnancies and ensuring maternal and neonatal safety, with its appropriate application playing a key role in improving perinatal outcomes (1). However, as global cesarean section rates continue to increase, optimizing surgical scheduling and improving healthcare resource utilization while ensuring safety have become important issues in obstetric clinical practice (2, 3). In recent years, whether the timing of surgery (e.g., working days vs. non-working days) affects perinatal outcomes has gained widespread attention. Some studies have suggested that cesarean sections performed on weekends or holidays may be associated with an increased risk of adverse neonatal outcomes—the so-called “weekend effect” (4). A recent multicenter prospective cohort study by Yuan et al. (4) involving 9,097 cesarean deliveries in China reported that weekend delivery was associated with significantly increased risks of preterm birth, low birth weight, and neonatal intensive care unit admission. However, as the authors acknowledged, their study lacked detailed data on surgical indications and could not distinguish between elective and emergency procedures. Nevertheless, these observational studies often struggle to adequately control for key confounding factors such as surgical indications, urgency, and hospital resource allocation, which may render their conclusions controversial. Of note, previous research has primarily focused on emergency or unplanned cesarean sections or has analyzed all cesarean types collectively, making it difficult to accurately assess the safety of elective procedures performed on different days (5, 6). For scheduled cesarean sections, surgical timing can typically be arranged in advance by the clinical team based on maternal conditions and hospital scheduling, theoretically minimizing variability in care attributable to temporal factors. However, in practice, non-working day periods, including weekends and statutory holidays, may involve differences in staffing, laboratory support, and multidisciplinary collaboration capacity (7). Whether these differences substantively impact maternal and neonatal outcomes remains insufficiently supported by high-quality evidence. In recent years, propensity score matching (PSM) has been widely applied to control for confounding in observational studies, with its advantages demonstrated in comparisons of outcomes related to different delivery modes and surgical timing (8). By simulating the randomization process, this method effectively balances baseline differences between groups, providing a more reliable analytical approach for studies examining temporal factors. Therefore, this study focuses on scheduled cesarean sections for term singleton pregnancies and applies PSM to rigorously control for potential confounders. It aims to objectively evaluate the impact of scheduling surgery on working versus non-working days on key perinatal outcomes, including maternal postoperative complications, neonatal transfer requirements, intraoperative and postoperative outcomes, and umbilical cord arterial blood gas parameters. The findings are expected to provide evidence-based guidance for optimizing cesarean section scheduling practices and rationally allocating non-working day medical resources while further clarifying the role of “temporal effects” in elective obstetric surgery.

## Methods

### Study design and ethical approval

This single-center retrospective cohort study included women with term singleton pregnancies who underwent scheduled cesarean sections at the Second People’s Hospital of Nanning from January 2024 to December 2024, with surgeries scheduled between 08:00 and 18:00. The study protocol was reviewed and approved by the Ethics Committee of the Second People’s Hospital of Nanning. All data were anonymized, adhering to the ethical principles of the Declaration of Helsinki. Given the retrospective nature of the study and the anonymization of data, informed consent was waived by the ethics committee.

### Study population

The inclusion criteria were as follows: women with singleton pregnancies; gestational age ≥ 37 weeks; scheduled cesarean sections, defined as pre-scheduled, non-labor, non-emergency procedures; and complete clinical data.

**Figure 1:**
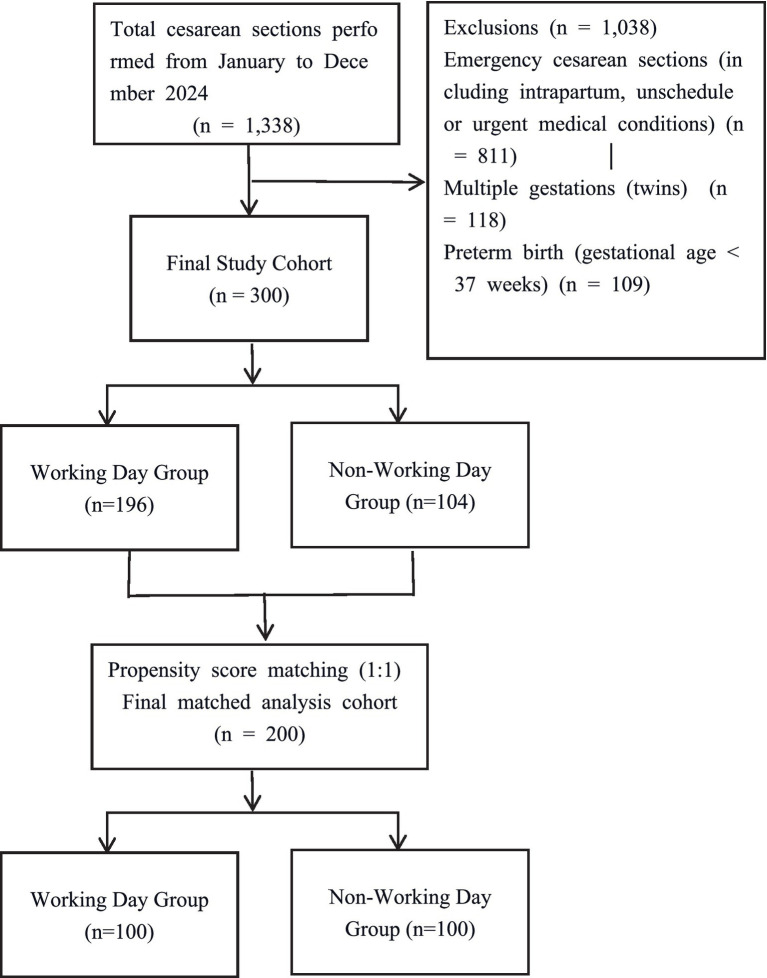
Flowchart of patient screening and enrollment.

The exclusion criteria were as follows: women with emergency cesarean sections or cesarean sections performed after labor onset; multiple pregnancies; gestational age < 37 weeks; concurrent severe medical or surgical conditions, such as heart disease, liver or kidney insufficiency, severe anemia, uncontrolled hypertension, or diabetes mellitus; fetuses with severe congenital anomalies; and missing clinical data affecting the analysis. The patient selection flowchart is presented in Figure 1.

### Grouping criteria

Participants were divided into two groups based on the surgery date.

#### Working-day group

Surgeries scheduled from Monday to Friday (excluding national holidays).

#### Non-working-day group

Surgeries scheduled on Saturdays, Sundays, or national holidays, excluding compensatory working days, i.e., weekend days that are officially designated as working days to adjust for holiday schedules.

### Surgical team configuration

To ensure consistent surgical quality, the scheduled cesarean section team at our center follows a standardized staffing configuration, which remains unaffected regardless of whether the procedure is performed on a working or non-working day. The day shift (08:00–18:00) staff is composed of:

#### Working day roster

The surgical team consists of 2 senior doctors (holding the title of attending physician or above and over 10 years of experience, taking turns managing surgeries on the day), 2 junior doctors (resident doctors who completed standardized residency training or attending physicians), and 2–3 doctors undergoing standardized residency training. All day shifts are succeeded by the next shift after 18:00.

#### Non-working day roster

The surgical team includes 1 senior doctor (with the same qualifications as the working-day senior doctors, on a continuous 24-h shift from 08:00 to 08:00 the following day, responsible for all surgeries and clinical decision-making), 2 junior doctors, and 2–3 doctors undergoing standardized residency training. Except for the senior doctor on a 24-h shift, other day shift staff are succeeded by the next shift after 18:00.

#### Night shift (18:00–08:00 the next day)

Irrespective of whether it is a working day or not, the night shift team is uniformly composed of 1 senior doctor, 1 junior doctor, and 1–2 doctors undergoing standardized residency training, responsible for emergency surgeries and inpatient ward management at night. Scheduled cesarean sections are not performed for the night shift.

All scheduled cesarean sections are performed under the above personnel configuration, with the senior doctor serving as the primary surgeon and the junior doctor as the first assistant. This staffing model ensures that the core experience level and team structure remain highly consistent, regardless of whether the surgery is scheduled on a working day or a non-working day, thus minimizing potential bias due to differences in human resources.

Although the number of senior physicians differs (two on working days versus one on a 24-h shift on non-working days), the senior physician on non-working days is the primary surgeon for all scheduled cesarean sections, with no reduction in attending-level presence during surgery. The 24-h shift may introduce fatigue; however, no scheduled cesarean sections are performed after 18:00, and the senior physician’s duty includes rest periods before surgery when elective cases are scheduled in the morning.

#### Anesthesia staffing

On working days, one senior anesthesiologist (≥10 years of experience), one junior anesthesiologist, and one resident undergoing standardized residency training are present during the daytime. On non-working days, the same configuration is maintained, with the senior anesthesiologist on a 24-h shift.

Neonatology staffing: The neonatology team (one attending physician or associate chief physician and one resident) is on 24-h standby and is called by the obstetrics team to the operating room as needed. No difference exists between working and non-working days.

#### Nursing staffing

The operating room nursing team consists of three experienced nurses (one circulating nurse and two scrub nurses) on both working and non-working days.

### Outcome measures

#### Baseline characteristics

Demographic characteristics included age, gravidity, parity, and the number of previous cesarean deliveries.

Perinatal indicators included gestational age at delivery, pre-pregnancy body mass index (BMI), gestational diabetes mellitus (GDM), and hypertensive disorders in pregnancy (HDP).

Surgical indications were categorized as scarred uterus, placenta previa, or other cesarean deliveries, including breech presentation, oligohydramnios, cephalopelvic disproportion, advanced maternal age [≥35 years], and severe preeclampsia as defined by the American College of Obstetricians and Gynecologists (ACOG) criteria.

Conception methods included natural conception and assisted reproductive technology.

#### Complete blood count indicators

Complete blood count indicators included preoperative hemoglobin (Hb), hematocrit (HCT), white blood cell count (WBC), and neutrophil percentage (NEU%).

They also included postoperative values of Hb, HCT, WBC, and NEU%.

#### Intraoperative and postoperative metrics

Intraoperative and postoperative metrics included operative duration, intraoperative blood loss, postoperative length of hospital stay, and duration of postoperative prophylactic antibiotic use (defined as the time from the end of surgery to the final prophylactic antibiotic dose, measured in hours).

#### Maternal and neonatal outcome indicators

Maternal outcomes included postoperative fever, postpartum hemorrhage, and poor incision healing.

Neonatal outcomes included neonatal transfer [to a neonatology department or neonatal intensive care unit (NICU)].

Umbilical cord arterial blood gas analysis was used to measure pH and lactate (Lac). Although umbilical cord blood gas values primarily indicate fetal status and intrapartum physiology, we have included them as a standard objective measure of neonatal well-being at delivery. In scheduled cesarean sections without labor, abnormal values may also indicate suboptimal intraoperative management (e.g., prolonged uterine incision-to-delivery interval or cord compression).

The following outcomes were defined prospectively:

*Postpartum hemorrhage*: estimated blood loss ≥1,000 mL during cesarean delivery (9, 10).
*Postoperative fever*: axillary temperature ≥37.3 °C measured on two separate occasions at least 6 h apart, excluding the first 24 h after surgery.
*Poor incision healing*: any of the following occurring within 30 days after surgery—wound dehiscence (≥1 cm separation), purulent drainage with surrounding erythema, or need for surgical incision and drainage.
*Neonatal transfer*: physical transfer of the neonate from the mother–baby unit to the neonatology department or neonatal intensive care unit (NICU) for any medical reason within 24 h of birth.

### Statistical analysis

**Figure 2:**
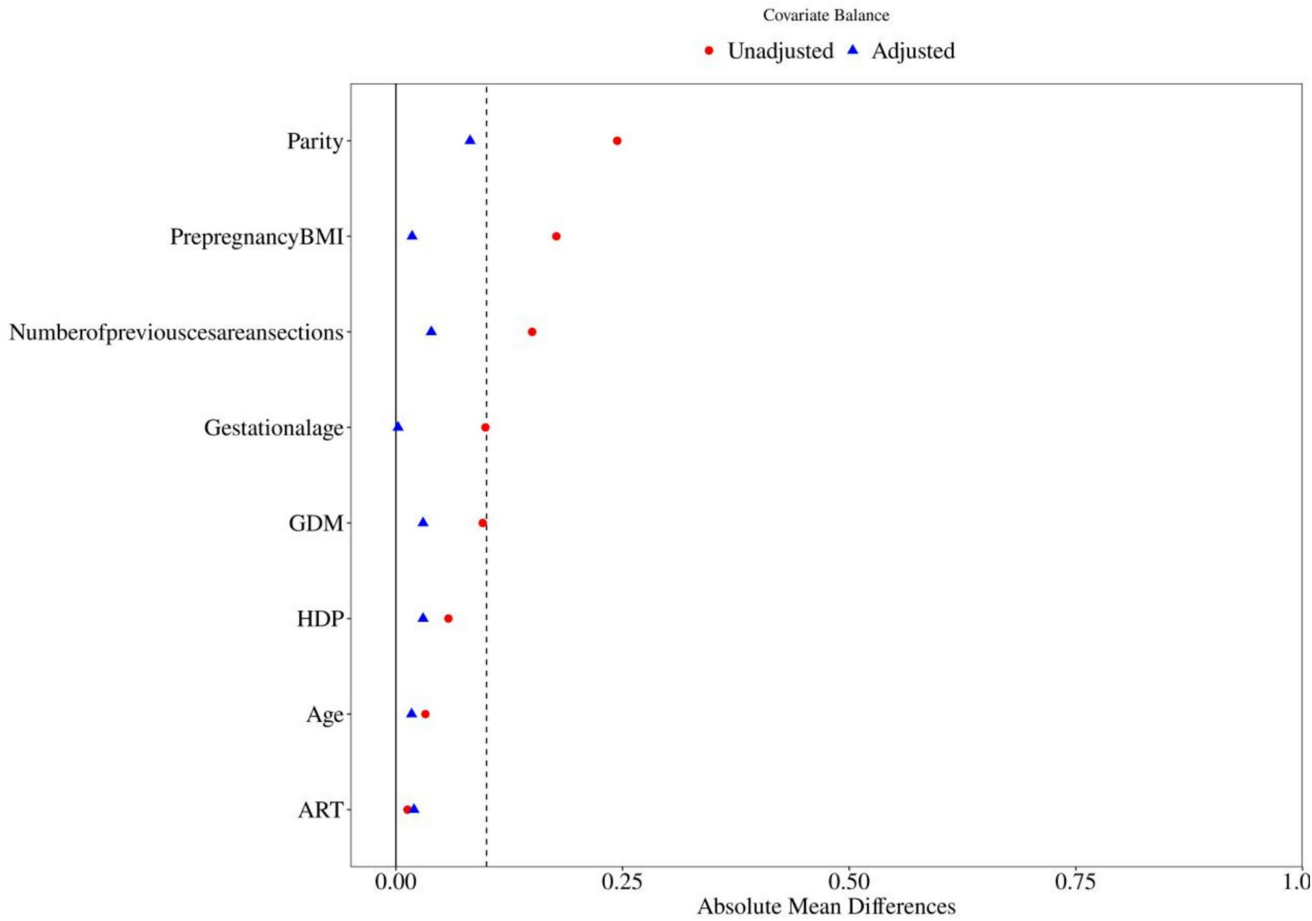
SMD plot.

**Figure 3:**
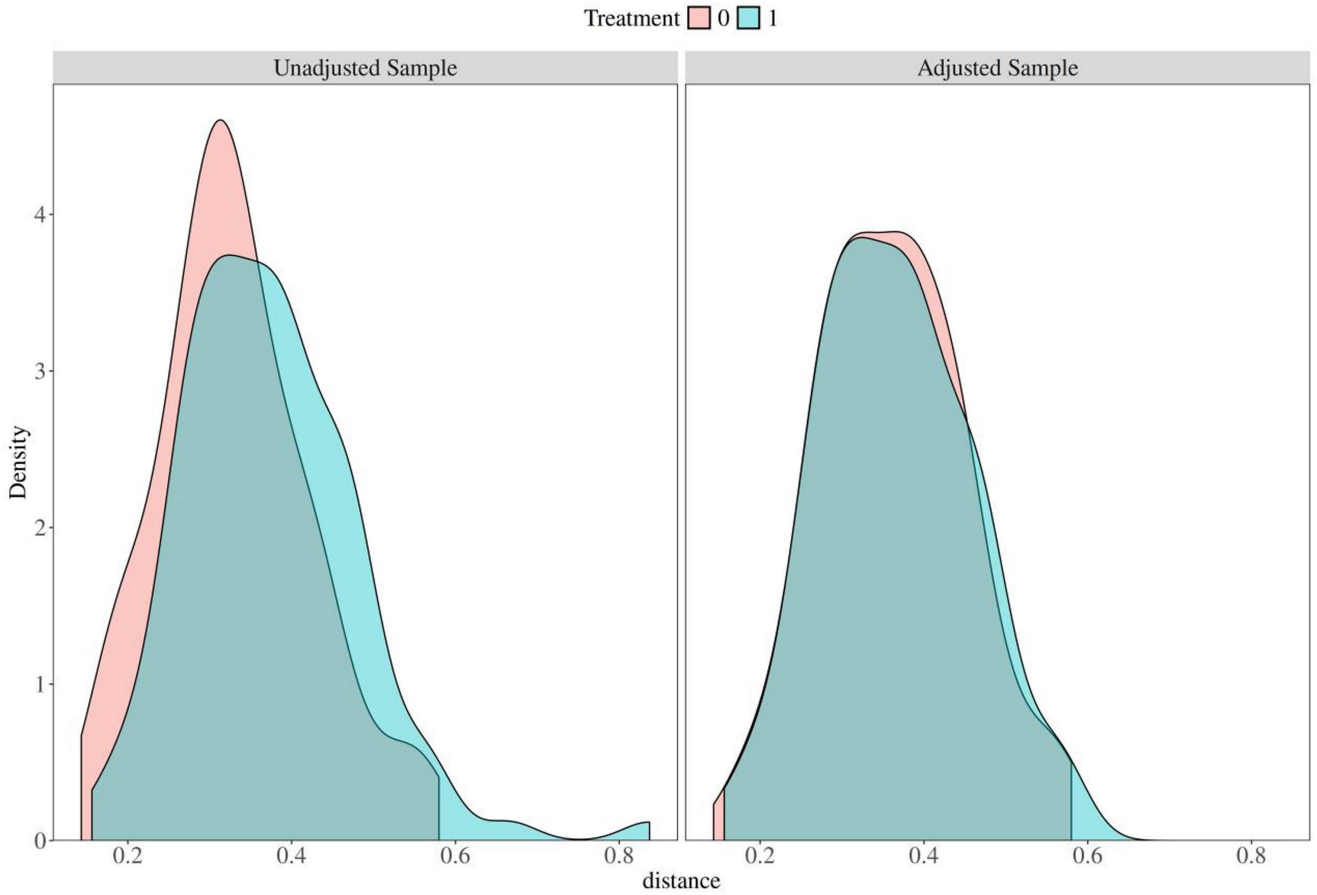
Density plot.

Statistical analyses were performed using SPSS (version 29.0), Zstats 1.0,^1^ the Storm platform,^2^ and R version 4.3.3. Normally distributed continuous variables were presented as mean ± standard deviation ( $\bar{x}$ ± *s*), while non-normally distributed continuous variables were presented as median (first quartile and third quartile). Categorical variables were presented as frequencies (percentages). For comparisons between groups, the independent samples t-test was used for normally distributed continuous variables, the Mann–Whitney U test was used for non-normally distributed continuous variables, and the chi-squared test or Fisher’s exact test was used for categorical variables, as appropriate. To control for confounding bias between the working day and non-working-day groups, PSM was used for balancing. Propensity scores were calculated using a logistic regression model with surgery group (non-working day vs. working day) as the dependent variable and the following covariates: age, gestational age at delivery, parity, number of previous cesarean deliveries, conception method, HDP, GDM, and pre-pregnancy BMI. Surgical indication was not included as a matching variable because it is a consequence of the underlying clinical conditions (e.g., scarred uterus or malpresentation), and its distribution was already balanced between the groups before matching (*p* = 0.922). Moreover, its inclusion could induce overadjustment bias as it lies on the causal pathway from maternal characteristics to the decision of scheduling. Matching was performed using 1:1 nearest neighbor matching without replacement, with a caliper width set to 0.2 times the standard deviation of the propensity score. The caliper width was set to 0.2 times the standard deviation of the logit of the propensity score, as recommended by Austin (2011) to balance bias and precision. After matching, covariate balance was assessed using the standardized mean difference (SMD), with an SMD < 0.1 indicating adequate balance between the groups. Balance diagnostics are presented in Figure 2 (SMD plot) and Figure 3 (density plot). To account for potential residual confounding after PSM, multivariable linear regression models were further used to evaluate the independent associations between surgery scheduling day (non-working day vs. working day) and intraoperative and postoperative metrics, as well as umbilical cord blood gas parameters. Separate linear regression models were constructed with operative duration, intraoperative blood loss, postoperative length of hospital stay, duration of postoperative prophylactic antibiotic use, umbilical cord arterial pH, and lactate level as dependent variables. The grouping variable (non-working day = 1, working day = 0) served as the primary independent variable, with adjustment for age, gestational age at delivery, parity, number of previous cesarean deliveries, conception method, GDM, HDP, and pre-pregnancy BMI as covariates. After PSM, a multivariable linear regression analysis was performed to adjust for residual confounding. Regression assumptions were tested, and no serious violations were detected. Matched-pair dependence was accounted for using generalized estimating equations. The results are presented as unstandardized regression coefficients (*β*) with their 95% confidence intervals (CIs). Multicollinearity among independent variables was assessed using the variance inflation factor (VIF), with VIF < 5 indicating no significant collinearity. A two-sided *p*-value of < 0.05 was considered statistically significant.

## Results

### Comparison of baseline characteristics

Before matching, no statistically significant differences were observed between the two groups in age, pre-pregnancy BMI, conception method, HDP, GDM, or number of previous cesarean deliveries (*p* > 0.05). However, gravidity and parity were significantly higher in the non-working-day group than in the working-day group (gravidity: *p* = 0.035; parity: *p* = 0.027), indicating the presence of confounding bias. After matching, all matching variables (age, pre-pregnancy BMI, gravidity, parity, conception method, HDP, GDM, and the number of previous cesarean deliveries) showed no statistically significant differences between the two groups (*p* > 0.05), and the absolute values of standardized mean differences (SMDs) were all less than 0.1, indicating good baseline balance between the groups. Common support was assessed by examining the overlap of propensity score distributions between the groups; no patients were discarded due to non-overlap. Surgical indications were categorized as scarred uterus, placenta previa, or other primary cesarean deliveries, including breech presentation, oligohydramnios, cephalopelvic disproportion, advanced maternal age, and severe preeclampsia, with a balanced distribution between the groups both before (*p* = 0.922) and after matching (*p* > 0.99) (Table 1).

**Table 1:** Comparison of baseline characteristics between the groups.

Variable	Pre-PSM (*n* = 300)				Post-PSM (*n* = 200)			
	Working-day group (*n* = 196)	Non-working-day group (*n* = 104)	*p*	SMD	Working-day group (*n* = 100)	Non-working-day group (*n* = 100)	*p*	SMD
Age (years)	33.54 ± 4.84	33.38 ± 5.17	0.783	−0.032	33.55 ± 4.94	33.46 ± 5.20	0.900	−0.017
Pre-pregnancy BMI (kg/m^2^)	22.07 (20.54, 24.91)	23.01 (21.20, 25.83)	0.100	0.177	23.21 (21.22, 26.52)	22.95 (21.21, 25.45)	0.780	−0.018
Gravidity	3.00 (2.00, 4.00)	3.00 (2.00, 4.00)	0.035	0.212	3.00 (2.00, 4.00)	3.00 (2.00, 4.00)	0.291	0.103
Parity	1.00 (0.00, 1.00)	1.00 (1.00, 2.00)	0.027	0.260	1.00 (0.00, 1.25)	1.00 (1.00, 2.00)	0.469	0.086
Gestational age (days)	271.00 (267.00, 274.00)	270.50 (266.00, 274.00)	0.894	−0.099	270.58 ± 5.43	270.64 ± 6.30	0.943	0.010
ART, *n* (%)	29 (14.80)	14 (13.46)	0.754	−0.039	15 (15.00)	13 (13.00)	0.684	−0.059
HDP, *n* (%)	19 (9.69)	16 (15.38)	0.144	0.158	11 (11.00)	14 (14.00)	0.521	0.086
GDM, *n* (%)	66 (33.67)	25 (24.04)	0.084	−0.225	22 (22.00)	25 (25.00)	0.617	0.069
Number of previous cesarean sections	1.00 (0.00, 1.00)	1.00 (0.00, 1.00)	0.220	0.158	1.00 (0.00, 1.00)	1.00 (0.00, 1.00)	0.794	0.039
Surgical indication			0.922				>0.99	
Scarred uterus, *n* (%)	124 (63.27%)	68(65.38%)		—	65 (65.00%)	65 (65.00%)		—
Placenta previa, *n* (%)	3 (1.53%)	1 (0.97%)		—	1 (1.00%)	1 (1.00%)		—
Other primary cesarean section, *n* (%)	69 (35.20%)	35 (33.65%)		—	34 (34.00%)	34 (34.00%)		—

BMI, body mass index; ART, assisted reproductive technology; HDP, hypertensive disorders of pregnancy; GDM, gestational diabetes mellitus; PSM, propensity score matching.

### Comparison of complete blood count indicators

Before matching, no significant differences were observed between the two groups in preoperative Hb, HCT, WBC, and NEU% and in postoperative Hb, HCT, WBC, and NEU% (*p* > 0.05). After matching, these indicators still showed no statistically significant differences between the groups (*p* > 0.05), with absolute standardized mean differences (SMDs) all < 0.1, indicating that the complete blood count indicators were well balanced and comparable between the two groups (Table 2).

**Table 2:** Comparison of complete blood count indicators between the groups.

Variable	Pre-PSM (*n* = 300)				Post-PSM (*n* = 200)			
	Working-day group (*n* = 196)	Non-working-day group (*n* = 104)	*p*	SMD	Working-day group (*n* = 100)	Non-working-day group (*n* = 100)	*p*	SMD
Preoperative								
HB (g/L)	120.18 ± 11.87	119.27 ± 12.34	0.532	−0.074	119.88 ± 12.27	119.45 ± 12.46	0.806	−0.035
HCT	0.37 ± 0.03	0.36 ± 0.03	0.322	−0.117	0.37 ± 0.03	0.36 ± 0.03	0.504	−0.094
WBC (×10^9^/L)	8.75 ± 2.09	8.69 ± 2.03	0.804	−0.031	8.80 (7.27, 10.30)	8.30 (7.38, 9.95)	0.627	−0.047
NEU%	0.73 (0.70, 0.77)	0.72 (0.69, 0.76)	0.471	0.005	0.73 (0.70, 0.76)	0.72 (0.69, 0.76)	0.528	0.004
Postoperative								
HB (g/L)	114.03 ± 12.10	112.29 ± 12.80	0.246	−0.136	115.07 ± 12.67	112.94 ± 12.57	0.234	−0.169
HCT	0.35 ± 0.03	0.34 ± 0.04	0.111	−0.185	0.35 ± 0.03	0.35 ± 0.03	0.112	−0.226
WBC (×10^9^/L)	12.10 (10.70, 14.10)	12.45 (10.70, 15.33)	0.367	0.111	12.20 (10.78, 14.20)	12.45 (10.78, 15.33)	0.512	0.065
NEU%	0.81 ± 0.04	0.81 ± 0.05	0.724	−0.042	0.81 ± 0.04	0.81 ± 0.04	0.790	−0.038

Hb, hemoglobin; HCT, hematocrit; WBC, white blood cell count; NEU, neutrophil percentage.

### Comparison of intraoperative and postoperative metrics

Before and after matching, no significant differences were observed between the two groups in operative duration, intraoperative blood loss, postoperative length of hospital stay, or duration of postoperative prophylactic antibiotic use (*p* > 0.05) (Table 3).

**Table 3:** Comparison of intraoperative and postoperative metrics between the groups.

Variable	Pre-PSM (*n* = 300)				Post-PSM (*n* = 200)			
	Working-day group (*n* = 196)	Non-working-day group (*n* = 104)	*p*	SMD	Working-day group (*n* = 100)	Non-working-day group (*n* = 100)	*p*	SMD
Operative duration (min)	59.00 (47.00, 67.00)	55.00 (47.00, 64.00)	0.071	−0.294	58.50 (49.00, 68.00)	55.00 (47.00, 64.00)	0.070	−0.322
Intraoperative blood loss (mL)	300.00 (300.00, 400.00)	350.00 (300.00, 400.00)	0.149	0.144	300.00 (300.00, 400.00)	400.00 (300.00, 400.00)	0.489	0.078
Postoperative length of hospital stay (days)	5.00 (5.00, 6.00)	5.00 (5.00, 6.00)	0.184	−0.165	5.00 (5.00, 6.00)	5.00 (5.00, 6.00)	0.136	−0.229
Duration of postoperative prophylactic antibiotic use (h)	24.00 (24.00, 24.00)	24.00 (24.00, 24.00)	0.350	−0.344	24.00 (24.00, 24.00)	24.00 (24.00, 24.00)	0.175	−0.650

### Comparison of maternal and neonatal outcomes

Before matching, the incidence of postoperative fever (working-day group: 2 cases, 1.02%; non-working-day group: 0 cases) and neonatal transfer (working-day group: 3 cases, 1.53%; non-working-day group: 0 cases) was very low in both groups, with no statistically significant differences (*p* > 0.05). Umbilical cord arterial blood gas analysis showed no significant differences in pH or lactate levels between the two groups (*p* > 0.05).

After matching, no significant differences were observed between the groups in postoperative fever [1.0% vs. 0%; risk difference 1.0% (95% CI: −0.9 to 2.9%); *p* = 1.000], neonatal transfer [2.0% vs. 0%; risk difference 2.0% (95% CI: −0.7 to 4.7%); *p* = 0.497], or umbilical cord arterial pH [median difference 0.000 (95% CI: −0.020 to 0.010); *p* = 0.836] and lactate levels [median difference 0.100 (95% CI: −0.100 to 0.300); *p* = 0.406] (Table 4).

**Table 4:** Comparison of maternal and neonatal outcomes between the groups.

Variable	Pre-PSM (*n* = 300)				Post-PSM (*n* = 200)				
	Working-day grou*p* (*n* = 196)	Non-working-day group (*n* = 104)	*p*	SMD	Working-day group (*n* = 100)	Non-working-day group (*n* = 100)	*p*	SMD	(95% CI)
Postoperative fever, *n* (%)	2 (1.02)	0 (0.00)	0.546^a^	−0.126	1 (1.00)	0 (0.00)	1.000^a^	−0.142	(−0.9–2.9%)
Neonatal transfer, *n* (%)	3 (1.53)	0 (0.00)	0.5151	−0.154	2 (2.00)	0 (0.00)	0.497^a^	−0.202	(−0.7–4.7%)
Umbilical cord arterial blood gas									
pH	7.28 (7.25, 7.31)	7.28 (7.26, 7.32)	0.705	−0.032	7.28 (7.25, 7.31)	7.28 (7.26, 7.32)	0.836	0.062	(−0.020–0.010)
Lactate (Lac, mmol/L)	2.40 (2.00, 2.92)	2.30 (2.00, 2.90)	0.712	−0.166	2.40 (2.08, 2.90)	2.25 (2.00, 2.90)	0.406	−0.123	(−0.100–0.300)

Lac, lactate; PSM, propensity score matching. No case met the definition of postpartum hemorrhage.

a Fisher’s exact test was used where indicated.

### Multivariable linear regression analysis

After adjusting for confounding factors, non-working day surgery was independently associated with a shorter operative duration (*β* = −3.82, 95% CI: −7.22 to −0.42, *p* = 0.029). This difference of approximately 3.8 min is statistically significant but unlikely to be clinically significant, as it falls below the typical threshold for operative duration considered relevant to patient outcomes. No significant differences were observed between the two groups in the remaining outcomes: intraoperative blood loss (*β* = 1.01, 95% CI: −24.89 to 26.69, *p* = 0.939), postoperative length of hospital stay (*β* = −0.11, 95% CI: −0.37 to 0.14, *p* = 0.394), duration of postoperative prophylactic antibiotic use (*β* = −1.54, 95% CI: −4.29 to 1.21, *p* = 0.275), umbilical cord arterial pH (*β* = 0.00, 95% CI: −0.02 to 0.02, *p* = 0.705), and lactate level (*β* = −0.03, 95% CI: −0.34 to 0.28, *p* = 0.849) (Table 5).

**Table 5:** Multivariable linear regression analysis: independent associations of non-working day surgery with surgical and blood gas outcomes.

Outcome	*β* (95% CI)	*p*
Operative duration (min)	−3.82 (−7.22 to −0.42)	0.029
Intraoperative blood loss (mL)	1.01 (−24.89–26.69)	0.939
Postoperative length of hospital stay (days)	−0.11 (−0.37–0.14)	0.394
Duration of postoperative prophylactic antibiotic use (h)	−1.54 (−4.29–1.21)	0.275
Umbilical cord arterial pH	0.00 (−0.02–0.02)	0.705
Umbilical cord arterial lactate (mmol/L)	−0.03 (−0.34–0.28)	0.849

Adjusted for confounding factors including age, gestational age, parity, number of previous cesarean sections, conception method, GDM, HDP, and pre-pregnancy BMI. β, unstandardized regression coefficient; CI, confidence interval; GDM, gestational diabetes mellitus; HDP, hypertensive disorders of pregnancy; BMI, body mass index.

## Discussion

This study systematically assessed the impact of performing scheduled cesarean sections for term singleton pregnancies on non-working days and working days on maternal and neonatal outcomes, using propensity score matching to rigorously control for confounding factors. The results demonstrated that, in settings with adequate staffing and standardized procedures, performing scheduled cesarean sections on non-working days was not associated with worse key perinatal outcomes, such as postoperative fever, neonatal transfer rate, surgical blood loss, length of hospital stay, duration of postoperative prophylactic antibiotic use, or umbilical cord blood gas parameters. Conversely, the multivariable regression analysis indicated that surgery performed on non-working days was independently associated with a shorter operative duration (*β* = −3.82, 95% CI: −7.22 to −0.42). This finding challenges the assumption that the “weekend effect” applies universally to elective surgeries and provides empirical evidence for optimizing cesarean section scheduling strategies. Numerous prior studies have reported the “weekend effect,” in which patients admitted or undergoing surgery on weekends or holidays face a higher risk of adverse outcomes. For example, Liu et al. (11) conducted a retrospective analysis of 74,664 patients undergoing various surgical procedures and found that those who had surgery on non-working days experienced significantly higher in-hospital mortality (2.6% vs. 1.7%) and postoperative major organ failure rates (3.5% vs. 2.3%) than the working-day group, along with longer operative times, greater blood loss, and a lower proportion of minimally invasive surgery. However, such studies largely encompass emergency and unplanned procedures, making direct extrapolation to scheduled cesarean sections challenging. For scheduled cesarean sections, the availability of thorough preoperative assessment, the ability to schedule surgical teams in advance, and greater control over resource allocation may potentially weaken or even negate the traditional “weekend effect.”

A recent multicenter study by Yuan et al. (4) reported that weekend cesarean delivery was associated with increased risks of preterm birth, low birth weight, and NICU admission in 9,097 Chinese women. However, as the authors acknowledged, their study lacked data on surgical indications and could not distinguish elective from emergency procedures. In contrast, our study focused specifically on scheduled cesarean sections and achieved excellent baseline balance through propensity score matching, including surgical indications. Moreover, our institution maintains consistent surgical team staffing across working and non-working days. These differences likely explain why we did not observe a “weekend effect” in our study population.

After controlling for confounding factors, although the shorter operative duration associated with non-working-day surgery was statistically significant, this difference was minimal and not clinically significant. We hypothesize that this subtle difference may be attributed to the lower surgical volume on non-working days and more efficient surgical turnover. Literature has suggested a tendency in clinical practice to prioritize complex or high-risk cases on working days, while relatively straightforward scheduled cesarean sections may be scheduled on weekends (12). An analysis of surgical characteristics in public hospitals has revealed that patients undergoing weekend surgery had significantly shorter median hospital stays (3.0 days vs. 5.0 days) and preoperative hospital stays (0.5 days vs. 1.5 days) than those undergoing weekday surgery (13), suggesting that non-working day surgery may offer efficiency advantages in resource allocation and operational flow. In our institution, scheduled cesarean sections are performed by senior physicians regardless of the day. However, the observed shorter operative duration on non-working days is more likely attributed to system-level factors: a lower surgical volume reduces queueing and turnover time, standardized protocols ensure consistency, and the single senior physician on 24-h shift may have uninterrupted familiarity with the operating room environment. Seniority alone does not eliminate human error, but a combination of stable staffing, standardized workflows, and lower case load may improve efficiency without compromising safety. Previous research has shown a significantly higher proportion of emergency surgeries (14.7% vs. 6.8%) and organ transplant surgeries (7.1% vs. 4.2%) in non-working-day groups (11); another study has also found that the proportion of grade 4 surgeries was lower on weekends than on weekdays (28.9% vs. 37.0%) (13). Although the present study balanced key variables such as the number of previous cesarean deliveries and pregnancy complications through PSM, the potential residual influence of such “case-mix bias” cannot be completely excluded. The shorter operative duration on non-working days, while statistically significant, is of negligible clinical magnitude. It may reflect lower case volume and fewer interruptions; however, no practical recommendations should be based on this isolated finding.

Regarding maternal and neonatal safety, this study found no disadvantages in the non-working-day group concerning postoperative infection (e.g., fever), neonatal transfer, or cord blood acidosis. This is consistent with the findings of several international studies. One study using data from the state of Oregon in the United States revealed that after implementing a policy restricting early elective deliveries, adjustments in the timing of scheduled cesarean sections did not lead to a significant increase in neonatal morbidity (14). Additionally, research has suggested that as long as standardized operative protocols are adhered to (e.g., preoperative antibiotic prophylaxis and standardized hemostasis techniques), the perioperative risks of scheduled cesarean sections can be effectively managed, regardless of the day of surgery (6). Notably, the results of this study do not refute the presence of a “weekend effect” in emergency cesarean sections. In fact, studies have clearly shown that emergency cesarean sections performed on weekends are associated with higher rates of adverse perinatal outcomes, likely due to the urgency of the condition, time constraints in decision-making, and limitations in resource mobilization (4). Therefore, distinguishing between “elective” and “unplanned/emergency” cesarean sections is crucial. This study was strictly confined to “elective” procedures—defined as planned surgeries without labor or urgent medical indications—and its findings should not be extrapolated to emergency contexts.

From a broader surgical perspective, recent studies have also questioned the universality of the “weekend effect.” For example, when hospitals establish comprehensive weekend surgical support systems (e.g., dedicated anesthesia teams, ICU coverage, and rapid pathology services), the “weekend effect” can be significantly reduced or even eliminated (15). The increased risk of adverse outcomes for non-working day surgeries may be attributed to a lower proportion of senior physicians (1:3 on working days vs. 1:6 on non-working days), staff fatigue, and delayed responses from ancillary departments (11). We acknowledge that the staffing is not identical: non-working days have one senior physician on a 24-h call, which could theoretically increase fatigue. However, all scheduled cesarean sections on non-working days are performed during daytime hours (08:00–18:00) before significant fatigue accumulates, and no difference in outcomes was observed. This suggests that, under our institutional protocol, the staffing difference did not result in measurable harm.

### Limitations

This study has several limitations. It is a single-center retrospective study with a limited sample size (100 patients per group after matching). No formal sample size calculation was performed because of the retrospective nature. *Post-hoc* power analysis using G*Power (z-test for two independent proportions) with observed event rates (postoperative fever 1% vs. 0%, neonatal transfer 2% vs. 0%) showed that with 100 patients per group, this study had approximately 17% power to detect a 1% absolute difference in fever (*α* = 0.05) and 29% power to detect a 2% difference in transfer. Thus, this study was substantially underpowered to detect clinically significant differences in these rare outcomes. The possibility of type II error cannot be excluded, and non-significant findings should not be interpreted as evidence of a safety equivalence. The absence of postpartum hemorrhage in our cohort, while consistent with the low-risk profile of scheduled cesarean sections (mean prior cesarean ~1, no placenta accreta spectrum), is lower than published rates (typically 1–4%). This may reflect the exclusion of high-risk comorbidities and the single-center’s rigorous surgical technique, and the limited sample size also precludes detection of rare events. Long-term follow-up outcomes (e.g., maternal psychological status and quality of parent–infant bonding) were not assessed. This study evaluated only short-term in-hospital outcomes. ICU admission, hospital readmission, reoperation, and thromboembolic events were important maternal outcomes that were not evaluated. Five-minute Apgar scores, respiratory complications (e.g., transient tachypnea of the newborn and respiratory distress syndrome), NICU length of stay, and composite neonatal morbidity were important neonatal outcomes that were not evaluated. The omission of these outcomes limits a comprehensive safety assessment. Although the study period covered the entire year of 2024, it remains a short-term observation that may not reflect seasonal variations or the impact of policy changes. Despite the balanced distribution of surgical indications between the two groups, the “other primary cesarean section” category encompassed indications with diverse pathophysiological mechanisms (e.g., breech presentation and severe preeclampsia), and this internal heterogeneity may have potentially influenced the outcomes. Despite PSM, residual confounding may persist due to unmeasured variables, including the severity of cesarean indication (e.g., degree of placenta previa and estimated fetal weight in suspected macrosomia), the presence of placental abnormalities other than previa, fetal growth restriction, detailed maternal comorbidity burden (e.g., well-controlled and poorly controlled diabetes), provider preference for scheduling, and patient preference for delivery date. Propensity score matching cannot address unmeasured confounding, and causal inference should be made cautiously. The mean number of prior cesarean sections was approximately one in both groups, indicating that our study population predominantly consisted of women undergoing their first or second repeat cesarean. This limits generalizability to patients with multiple prior cesarean deliveries (≥3) who may have higher risks of adhesions, uterine rupture, and hemorrhage. Furthermore, patients with severe medical or surgical comorbidities (e.g., heart disease and advanced liver or renal insufficiency) were excluded from the study. Such patients may benefit more from weekday multispecialty support, and our findings should not be extrapolated to them. The staffing differences between working and non-working days could be most consequential in these complex cases. Finally, although we observed no differences in maternal or neonatal outcomes for scheduled cesarean sections performed on non-working days, this study did not evaluate the potential impact of adding scheduled cases on weekends to the management of concurrent emergency or intrapartum cases. Under our staffing model, which relies on a single 24-h in-house physician on non-working days, it remains unclear whether an increased volume of elective procedures could affect the timeliness or outcomes of emergency cesarean sections or intrapartum emergencies that may occur simultaneously. Future studies should specifically examine resource competition and its potential clinical consequences. Larger, multicenter studies are needed to enable stratified analyses.

## Conclusion

Within the available sample size and under the specific staffing conditions of this single-center study, no statistically significant differences in short-term maternal or neonatal outcomes were detected between scheduled cesarean sections performed on working and non-working days. These findings should be considered hypothesis-generating and require confirmation in larger, adequately powered studies.

### Future directions

Large-scale, multicenter prospective cohort studies are needed to further validate the generalizability of these findings and to explore the safety boundaries and optimal practice models for non-working day surgeries across different healthcare systems.

## Data Availability

The original contributions presented in the study are included in the article/supplementary material, further inquiries can be directed to the corresponding authors.

## Footnotes

1
www.zstats.net

2
www.medsta.cn/software
